# A network pharmacology–guided multi-omics and spatial single-cell framework nominates WT1 as a spironolactone-linked immune biomarker in prostate cancer

**DOI:** 10.3389/fphar.2026.1770261

**Published:** 2026-03-06

**Authors:** Zhen Zhu, Xingjun He

**Affiliations:** Department of Urology, Affiliated Hospital of Yangzhou University, Yangzhou, China

**Keywords:** prostate cancer, single-cell, spironolactone, tumor microenvironment, WT1

## Abstract

**Introduction:**

Spironolactone (SPI), a mineralocorticoid receptor antagonist with anti-androgenic activity, has emerged as a candidate for drug repurposing in prostate cancer (PCa). However, the cellular pharmacology governing its impact on the tumor microenvironment and specific molecular targets remains incompletely understood. This study aims to elucidate the regulatory circuitry linking SPI to immune modulation and tumor suppression in the PCa ecosystem.

**Methods:**

We employed an integrated framework combining network pharmacology with bulk, single-cell (scRNA-seq), and spatial transcriptomics (ST) to prioritize SPI-associated targets and map their microenvironmental context. The clinical and immunological relevance of the top candidate, Wilms Tumor 1 (WT1), was assessed using TCGA and GEO datasets. Finally, molecular docking and *in vitro* gain-of-function assays (proliferation, migration, clonogenicity, and apoptosis) in DU145 and PC-3 cell lines were conducted to validate functional mechanisms.

**Results:**

Network pharmacology identified WT1 as a central regulatory node in the SPI-PCa interaction network. In clinical cohorts, WT1 was significantly downregulated in tumor tissues compared to normal prostate; however, preserved high WT1 expression correlated with improved disease-free survival. Crucially, WT1-high tumors exhibited an “immune-hot” phenotype characterized by enhanced T-cell infiltration and antigen presentation pathways. scRNA-seq and ST analyses revealed that WT1 is heterogeneously expressed across malignant and stromal compartments, localizing to specific immune-interacting niches. Molecular docking suggested a potential structural compatibility between SPI and WT1, although direct binding remains to be experimentally confirmed. Functionally, restoring WT1 expression in PCa cells potently suppressed malignant behaviors, inhibiting proliferation and migration while triggering apoptosis.

**Conclusion:**

This study defines a novel SPI-WT1 axis, positioning WT1 as a druggable, immune-correlated biomarker in prostate cancer. By linking SPI pharmacology to WT1-associated microenvironmental features and tumor suppression, our findings provide a mechanistic rationale for repurposing Spironolactone as an immunomodulator to overcome therapeutic resistance in genitourinary oncology.

## 1 Introduction

Prostate cancer (PCa) exemplifies a persistent clinical paradox: substantial gains in screening and first-line treatment have not translated into durable control once disease becomes advanced. The major inflection point is therapeutic escape from androgen-axis suppression. Although androgen deprivation therapy (ADT) often produces an initial response, many patients ultimately develop castration-resistant prostate cancer (CRPC), a highly lethal stage with a median survival of less than 2 years (Le et al., 2023). This trajectory motivates intensified efforts to identify actionable molecular vulnerabilities and to accelerate therapeutic discovery through rational repurposing of approved agents.

One pragmatic repurposing candidate is spironolactone, a long-used mineralocorticoid (aldosterone) receptor antagonist and potassium-sparing diuretic routinely prescribed for hypertension and heart failure. Importantly, spironolactone also exerts anti-androgenic effects and is therefore used off-label in androgen-driven dermatologic disorders such as acne and hirsutism. Because androgen signaling remains a dominant driver of prostate tumorigenesis and progression, spironolactone has drawn growing attention as a potential anti-tumor agent. Population-based analyses have linked prolonged spironolactone exposure with a reduced risk of PCa (Bommareddy et al., 2022), and clinical reports highlight context-dependent effects and drug–drug interactions relevant to advanced PCa management (Sundar and Dickinson, 2012; Dhondt et al., 2019; Whyne et al., 2025). Nevertheless, the relevant downstream genetic targets and regulatory programs through which spironolactone might reshape PCa biology have not been comprehensively resolved.

Among candidate nodes that could connect drug action to tumor behavior, Wilms’ tumor gene 1 (WT1) stands out because of its multifaceted, context-dependent functions. WT1 encodes a zinc finger transcription factor originally characterized as a tumor suppressor in Wilms tumor (Hirahata et al., 2025; Nian et al., 2025), yet its role in adult malignancies remains heterogeneous and sometimes paradoxical. In leukemia and several solid tumors, WT1 is frequently overexpressed and can behave as an oncogenic driver; conversely, accumulating evidence indicates that WT1 may preserve tumor-suppressive roles in particular tissue contexts and microenvironmental states (Hirahata et al., 2025; Nian et al., 2025). In PCa specifically, the literature remains unsettled—reports have alternately implicated WT1 in metastatic promotion or suggested that diminished WT1 expression may facilitate tumor initiation—highlighting the need for integrative analyses that incorporate both tumor-intrinsic pathways and the surrounding microenvironment (Hirahata et al., 2025; Nian et al., 2025).

Against this backdrop, we pursued a workflow designed to couple drug-target prioritization with multi-omics interpretation of tumor context. We first implemented an AI-assisted network pharmacology strategy to rank spironolactone-associated candidates in PCa and identified WT1 as a top-scoring regulatory node. We then performed a multi-omics characterization of WT1 across bulk transcriptomic datasets, immune-activity and pathway-signature profiling, and public single-cell RNA-seq and Visium spatial transcriptomics to map its cellular sources and microenvironmental associations. To test functional relevance, we complemented computational analyses with WT1 restoration experiments in PCa cell lines, assessing proliferation, clonogenicity, migration, and apoptosis. Finally, molecular docking was used as a hypothesis-generating step to evaluate the structural plausibility of a spironolactone–WT1 interaction. Collectively, this integrated framework supports WT1 as an immune-associated biomarker and candidate therapeutic node in PCa and provides a rationale for further evaluating spironolactone repurposing.

## 2 Methods

### 2.1 Integrated computational screening for spironolactone-associated targets

An integrated *in silico* screening workflow was implemented to curate candidate targets linked to spironolactone and to contextualize them within prostate adenocarcinoma (PRAD). First, spironolactone-associated genes were retrieved from the Comparative Toxicogenomics Database (CTD; https://ctdbase.org/) by aggregating curated compound–gene interactions reported in the literature (Hopkins, 2008; Pushpakom et al., 2019). In parallel, target candidates were inferred using the Similarity Ensemble Approach (SEA; https://sea.bkslab.org/) based on chemical similarity profiles (Cheng et al., 2018), and the spironolactone structure was queried in SwissTargetPrediction (https://www.swisstargetprediction.ch/) to obtain ligand-based predictions (Pushpakom et al., 2019). Targets from CTD, SEA, and SwissTargetPrediction were consolidated and harmonized to official gene symbols to generate a non-redundant drug-associated set. To assemble a disease-relevant reference set, PRAD-associated genes were downloaded from GeneCards (https://www.genecards.org/) using “Prostate Adenocarcinoma (PRAD)” as the query term. Drug- and disease-derived lists were then intersected to prioritize shared candidates that could plausibly mediate spironolactone-relevant effects in PRAD; overlaps were visualized with a Venn diagram using SangerBox (http://www.sangerbox.com/), and the intersecting gene set was carried forward for network and downstream multi-omics analyses.

### 2.2 Network topology and functional enrichment profiling

To characterize functional connectivity among the intersecting candidates, a high-confidence protein-protein interaction (PPI) network was constructed in STRING (version 11.5; https://string-db.org/) with *Homo sapiens* specified and a minimum interaction confidence score of 0.9. Interaction edges were exported and imported into Cytoscape (version 3.8.2) for visualization and quantitative topology assessment. NetworkAnalyzer and related Cytoscape utilities were used to compute standard centrality metrics, including degree and betweenness centrality, and highly connected nodes were prioritized as putative hubs. In parallel, biological interpretation of the intersecting set was performed via Gene Ontology (GO) and Kyoto Encyclopedia of Genes and Genomes (KEGG) enrichment analyses using SangerBox. A hypergeometric test was applied, and terms with p < 0.05 were considered significantly enriched; results were summarized using bar plots and bubble plots to highlight dominant biological processes and pathways.

### 2.3 Clinical relevance and genomic landscape analysis

Clinical relevance of candidate genes was examined through bulk transcriptomic comparisons, survival modeling, and mutation–expression coupling analyses. For tumor–normal expression contrasts, TCGA-PRAD RNA-seq profiles were integrated with normal prostate expression from GTEx using TPM-formatted data preprocessed through the UCSC Xena Toil pipeline, and analyses were performed in SangerBox. Candidate expression levels were compared between TCGA tumor samples and GTEx normal tissues using appropriate parametric or non-parametric tests, with p < 0.05 indicating significance. Prognostic associations were evaluated using GEPIA2 (http://gepia2.cancer-pku.cn/), where patients were stratified by gene expression (top 30% versus bottom 30%) to generate Kaplan–Meier curves for overall survival and disease-free survival; significance was assessed by log-rank testing (p < 0.05) and effect size was reported as hazard ratios with 95% confidence intervals. To assess whether expression differences aligned with mutational status, mutation calls from TCGA-PRAD were categorized (e.g., DEL, INS, SNP) as previously described (Quigley et al., 2018). Genes with mutation frequencies >10% were tested for expression dependence on mutation status using the coin R package (independence_test), a non-parametric permutation framework; candidates meeting both mutation frequency >10% and p < 0.01 were retained and visualized using boxplots or violin plots.

### 2.4 Immune-cycle activity and pathway-level phenotyping

To relate candidate expression to the tumor immune context and pathway activity, immune-cycle profiling and pathway-level scoring were performed in complementary frameworks. Anti-tumor immune activity was quantified using the Tracking Tumor Immunophenotype (TIP) metaserver (v1.0) (Gong et al., 2024), which reports activity across the seven steps of the cancer–immunity cycle, spanning antigen release/presentation, priming, immune-cell trafficking and infiltration, recognition, and cytotoxic killing. RNA-seq data were uploaded to TIP with *H. sapiens* selected; TIP integrates approaches such as ssGSEA and CIBERSORT to estimate immune activity and immune-cell infiltration, and immune-cycle scores were compared between high- and low-expression groups for each candidate gene. In parallel, metabolic pathway activity was quantified using Gene Set Variation Analysis (GSVA) with KEGG-derived metabolic gene sets. Expression matrices were Z-score normalized prior to scoring, samples were stratified into high- and low-expression cohorts using the top and bottom 30% expression cutoffs, and the limma R package was used to identify pathways that differed significantly between groups for interpretation in the context of tumor metabolic reprogramming.

### 2.5 Single-cell and spatial transcriptomic mapping

Cell-type and tissue-context sources of WT1 were resolved using public single-cell RNA-seq and Visium spatial transcriptomics resources. For scRNA-seq, prostate cancer datasets (GSE141445 and GSE176031) were downloaded from GEO and processed in R using Seurat. After constructing Seurat objects from expression matrices and metadata, data were log-normalized (NormalizeData), highly variable features were identified (FindVariableFeatures), and scaled (ScaleData) (Li et al., 2024). Dimensionality reduction was performed with PCA (RunPCA) followed by UMAP embedding (RunUMAP) for visualization (Li et al., 2024). Cell clusters were annotated based on the labels reported in the originating studies and were corroborated using canonical marker genes. Given dropouts typical of single-cell assays, WT1 signal was visualized using Nebulosa, which applies gene-weighted kernel density estimation to recover sparse expression patterns. We acknowledge that this smoothing approach aims to visualize global trends rather than raw counts; therefore, normalized WT1 expression was aggregated; normalized WT1 expression was aggregated by cell type and compared using two-sided Kruskal–Wallis rank-sum testing (p < 0.05). For spatial transcriptomics, 10x Genomics Visium PRAD sections were obtained from the Sparkle database, including spot-level expression matrices and deconvolution-based cell-type composition estimates. Spot counts were normalized using Seurat NormalizeData (Ståhl et al., 2016; Marx, 2021), and WT1 distribution was visualized across sections using SpatialFeaturePlot. Spatial domains were annotated by the dominant cell type per spot (highest inferred proportion) and displayed using SpatialPlot; within each section, mean WT1 expression in each dominant microregion was calculated and Z-score standardized (scale) to facilitate cross-section comparisons, with summaries visualized as heatmaps using the fromto package (hplot1). Microenvironmental relationships were quantified by Spearman correlations across spatial spots, evaluating (i) pairwise correlations among inferred cell-type proportions and (ii) correlations between inferred cell-type proportions and WT1 expression; matrices and network graphs were generated with linkET, with correlation strength/direction encoded by color and significance annotated. To interrogate malignant versus non-malignant spatial heterogeneity, spots were stratified by malignant-cell proportion into malignant (proportion = 1), normal (proportion = 0), and mixed groups (0 < proportion <1); when malignant spots were sparse (<30), thresholds were adjusted (>0.5 as high malignancy, 0 as non-malignancy, remainder as low malignancy), and if group sizes remained insufficient, all spots with malignant-cell proportion >0 were merged and compared with the non-malignant group. Due to the non-independent nature of spatial spots, the Wilcoxon rank-sum test was applied to compare the distributions of expression values across all spots within defined regions, acknowledging that p-values may be inflated by spatial autocorrelation. Results were summarized with bar plots of mean expression and variability.

### 2.6 Cell culture models and gene expression quantification

DU145 and PC-3 human prostate cancer cell lines and the RWPE-1 normal prostate epithelial cell line were purchased from iCell Bioscience Inc. (Shanghai, China). The cell lines were recently authenticated using Short Tandem Repeat (STR) profiling (files available upon request) and tested negative for *mycoplasma* contamination. DU145 and PC-3 cells were maintained in DMEM (Gibco, United States) supplemented with 10% fetal bovine serum (FBS; Gibco, United States), 100 U/mL penicillin, and 100 μg/mL streptomycin. RWPE-1 cells were cultured in Keratinocyte Serum-Free Medium (K-SFM; Gibco, United States) supplemented with manufacturer-recommended growth factors. Cells were incubated at 37 °C in a humidified atmosphere with 5% CO2 and were used in logarithmic growth for all experiments. For downstream gene expression quantification, total RNA was extracted and WT1 mRNA levels were measured by qRT-PCR as follows.

For gene expression quantification, total RNA was isolated from cultured cells using the RNA-easy Total RNA Isolation Kit (Vazyme, Nanjing, China) according to the manufacturer’s instructions (Brett et al., 2013). For each sample, 1 μg RNA was reverse-transcribed into cDNA using the PrimeScript RT Reagent Kit (Takara, Japan) (Brett et al., 2013). Quantitative real-time PCR was carried out on a QuantStudio 5 Real-Time PCR System (Applied Biosystems, United States) using SYBR Green Master Mix (Beyotime, Shanghai, China) following standard procedures. GAPDH was employed as the internal reference gene, and relative expression was calculated using the 2^−ΔΔCT^ method. Primer sequences were:

GAPDH-F: CTC​CTC​CTG​TTC​GAC​AGT​CAG​C.

GAPDH-R: CCC​AAT​ACG​ACC​AAA​TCC​GTT.

WT1-F: AAT​GGA​CAG​AAG​GGC​AGA​GC.

WT1-R: ACA​CCG​TGC​GTG​TGT​ATT​CT.

### 2.7 Functional phenotypic assays: proliferation, migration, and apoptosis

#### 2.7.1 Cell viability

Cell viability was quantified using the Cell Counting Kit-8 (CCK-8; Dojindo, Japan). DU145 and PC-3 cells were seeded into 96-well plates at 3 × 103 cells per well and allowed to adhere overnight. After transfection with WT1 overexpression plasmids or vector control, cells were incubated for the indicated time points (0, 24, 48, 72, and 96 h). At each time point, 10 μL CCK-8 reagent was added to each well, followed by incubation at 37 °C for 2 h. Absorbance at 450 nm was recorded using a microplate reader (BioTek Instruments, United States). All experiments were performed in triplicate representing independent biological replicates.

#### 2.7.2 Clonogenicity

For colony formation, DU145 and PC-3 cells were trypsinized, counted, and plated into six-well plates at 800 cells per well. Cells were maintained for 10–14 days until colonies were visible, with medium replacement every 3 days. Colonies were washed with PBS, fixed in 4% paraformaldehyde for 20 min, and stained with 0.1% crystal violet for 30 min. After rinsing and air-drying, colonies containing more than 50 cells were photographed and quantified.

#### 2.7.3 Migration assessment

Migratory capacity was assessed by wound healing. DU145 and PC-3 cells were plated in six-well plates and cultured until approximately 90% confluence. A linear scratch was generated using a sterile 200-μL pipette tip, and initial scratch widths were visually inspected to ensure baseline consistency across wells. After washing twice with PBS to remove detached cells, serum-free medium was added to reduce proliferation-related closure. Wound images were captured at 0 h and at the indicated time points using an inverted microscope (Olympus, Japan). Wound width was quantified using ImageJ software (NIH, United States) by measuring the average gap distance at three distinct positions per well. Migration rate was calculated as the percentage of wound closure relative to baseline.

Apoptosis Analysis. Apoptosis was quantified using an Annexin V–FITC/PI apoptosis detection kit (Beyotime, Shanghai, China) according to the manufacturer’s protocol (Vermes et al., 1995; Galluzzi et al., 2009). DU145 and PC-3 cells transfected with WT1 overexpression plasmids or vector control were collected 48 h after transfection. Cells were washed twice with ice-cold PBS and resuspended in 1× binding buffer at 1 × 105 cells per 100 μL. Annexin V–FITC (5 μL) and propidium iodide (PI; 5 μL) were added, and samples were incubated for 15 min at room temperature in the dark. After addition of 400 μL binding buffer, samples were immediately analyzed on a BD FACSCanto II flow cytometer (BD Biosciences, United States). FlowJo software (Tree Star, United States) was used for analysis. A unified gating strategy was applied across all samples to ensure comparability, and total apoptosis was calculated as the sum of early and late apoptotic populations.

## 3 Results

### 3.1 Cross-database target convergence highlights WT1 as a central spironolactone-linked node in PRAD

**FIGURE 1:**
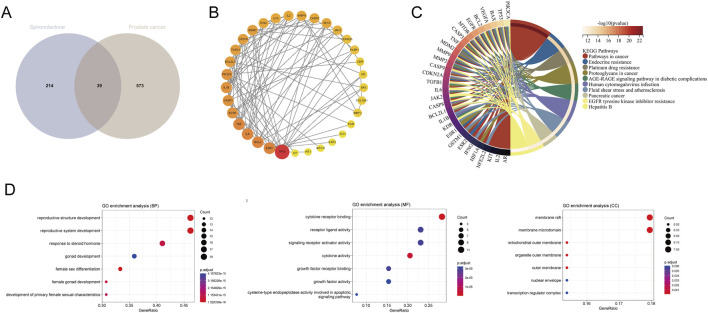
Candidate target nomination and enrichment overview. **(A)** Shown is the overlap between spironolactone-associated genes (214) and prostate cancer-related genes (573), yielding 39 shared candidates. **(B)** Connectivity among the 39 intersection genes is summarized as a STRING-derived protein-protein interaction (PPI) network. **(C)** KEGG enrichment results are presented as a chord diagram, emphasizing pathways including “Pathways in cancer” and “Endocrine resistance.” **(D)** GO term enrichment is displayed as bubble plots across Biological Process (BP), Molecular Function (MF), and Cellular Component (CC).

Putative spironolactone targets were assembled from the CTD, SEA, and SwissTargetPrediction resources, yielding 214 candidates. In parallel, 573 prostate cancer–associated genes were retrieved from GeneCards. Intersecting the two lists identified 39 shared targets (Figure 1A). A STRING-derived protein-protein interaction network built from these 39 proteins displayed dense connectivity; WT1 clustered at the network center together with TP53, TNF, IL6, and VEGFA and showed high degree centrality, consistent with a potential key regulatory position (Figure 1B). KEGG enrichment further indicated that the shared targets were significantly concentrated in tumor-relevant pathways, including “Pathways in cancer,” “Endocrine resistance,” “Proteoglycans in cancer,” and “Platinum drug resistance” (Figure 1C), aligning with programs involved in tumor development, endocrine therapy resistance, and cell survival. GO enrichment highlighted predominant associations with terms such as “reproductive structure/system development,” “response to steroid hormones,” “cytokine receptor binding,” and “membrane raft/microdomain” (Figure 1D). Collectively, these analyses support the interpretation that spironolactone may influence prostate cancer–related signaling networks linked to steroid hormone responses and cell fate regulation, with WT1 emerging as a representative core node.

### 3.2 Bulk cohort evidence indicates WT1 loss in tumors and links higher expression to improved disease control

**FIGURE 2:**
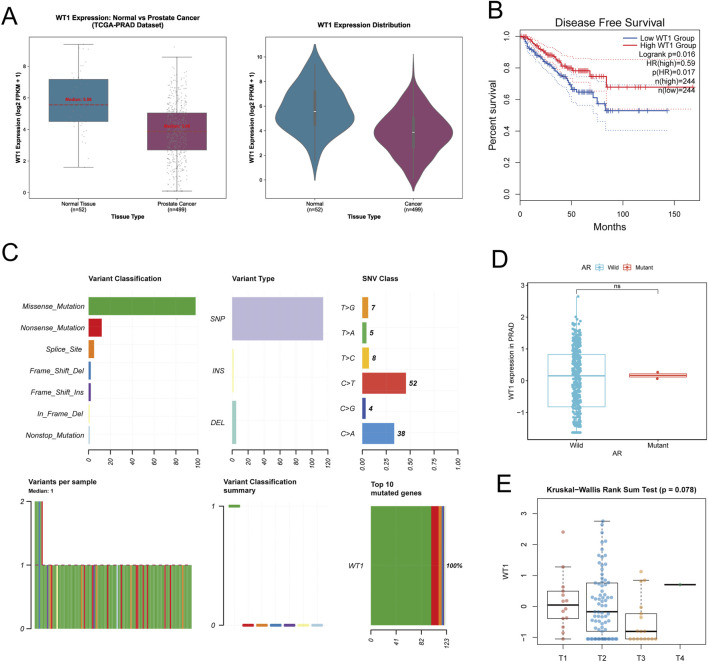
WT1 expression patterns, outcome association, and genomic context in prostate cancer. **(A)** WT1 transcript levels are contrasted between normal prostate (GTEx, n = 52) and PRAD tumors (TCGA-PRAD, n = 499) using box/violin plots, showing reduced expression in tumors. **(B)** Disease-Free Survival (DFS) in TCGA-PRAD is stratified by WT1 expression; improved DFS is observed in the WT1-high group (Log-rank p = 0.016). **(C)** The spectrum of WT1 genetic alterations is summarized by variant classification and single nucleotide variant (SNV) class. **(D)** WT1 expression is compared between Androgen Receptor (AR) wild-type and AR-mutant samples (ns). **(E)** WT1 expression across tumor stages (T1–T4) is shown, without a statistically significant stage-dependent difference (p = 0.078).

WT1 expression in prostate tumor versus normal tissue was examined by integrating TCGA-PRAD and GTEx datasets. WT1 was significantly reduced in tumor tissues compared with normal prostate tissues (Figure 2A), supporting a potential tumor-suppressive association in prostate cancer. For prognostic evaluation, TCGA-PRAD patients were stratified into high- and low-WT1 expression groups and assessed for disease-free survival (DFS). Improved DFS was observed in the high-expression group (Figure 2B), supported by log-rank test p = 0.016 and HR (high) = 0.59, indicating a reduced recurrence risk when WT1 expression is elevated. WT1 alterations in the TCGA cohort were then characterized. Mutations were uncommon and were mainly missense events, with SNPs representing the predominant mutation type (Figure 2C). This pattern suggests that WT1 functional disruption in prostate cancer is more likely driven by abnormal expression rather than frequent structural mutation. WT1 expression did not differ significantly between androgen receptor (AR) wild-type and AR-mutant samples (Figure 2D), implying limited dependence on AR mutation status. Likewise, stratification by T stage (T1–T4) showed no significant differences in WT1 expression (Kruskal–Wallis test p = 0.078, Figure 2E), consistent with WT1 downregulation occurring as an early molecular event rather than reflecting late-stage progression.

### 3.3 WT1-stratified tumors exhibit coordinated immune activation, metabolic rewiring, and attenuated malignancy signatures

**FIGURE 3:**
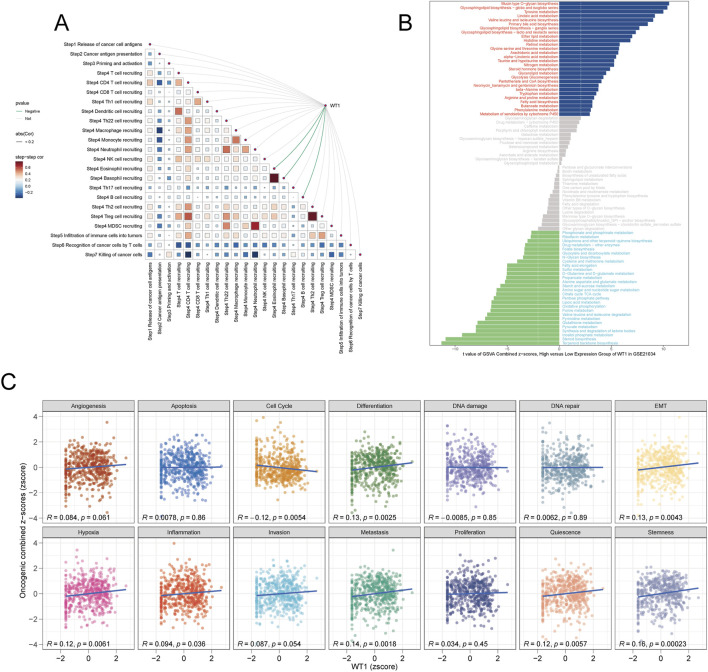
WT1-linked immune activity and metabolic pathway shifts. **(A)** Associations between WT1 expression and the seven-step cancer-immunity cycle are quantified with TIP, highlighting positive relationships with T cell recruitment and infiltration. **(B)** GSVA results depict differential enrichment of metabolic pathways, with amino acid metabolism favored in WT1-high tumors and energy/nucleotide metabolism predominating in WT1-low tumors. **(C)** Correlation scatter plots relate WT1 expression to cancer functional phenotype z-scores, including Cell Cycle, Invasion, Metastasis, and Apoptosis.

To evaluate immunological associations, the TIP platform was used to score the seven steps of the cancer immunity cycle in TCGA-PRAD samples and relate these scores to WT1 expression. WT1 levels were positively associated with multiple immune processes, including tumor antigen release and presentation (steps 1–2), T-cell priming and activation (step 3), recruitment and infiltration of CD4+/CD8+ T cells and NK cells (steps 4–5), and effector T cell–mediated tumor killing (step 7) (Figure 3A). These relationships indicate that higher WT1 expression aligns with a more active anti-tumor immune context. Because metabolic remodeling is tightly linked to tumor state, GSVA was performed on the GSE21034 dataset to compare metabolic pathway enrichment by WT1 expression group. In the WT1 high-expression group, pathways related to glycolipid and amino acid metabolism (e.g., glycosaminoglycan biosynthesis, valine/leucine/isoleucine biosynthesis, glycine/serine/threonine metabolism) were significantly upregulated. In contrast, the WT1 low-expression group exhibited greater activity of energy production and nucleotide synthesis pathways (e.g., TCA cycle, glycolysis, purine and pyrimidine metabolism, fatty acid metabolism) (Figure 3B). This pattern suggests that higher WT1 expression is associated with reduced engagement of highly proliferative metabolic programs. WT1 was also compared with z-scores representing malignant phenotypes. WT1 expression showed negative correlations with cell cycle progression, invasion, metastasis, proliferation, and stemness, while positive correlations were observed with cell differentiation and selected apoptosis-related phenotypes (Figure 3C). Together, these findings support an association between high WT1 expression and attenuated malignant behavior in prostate cancer.

### 3.4 Single-cell resolution defines WT1-enriched malignant and stromal subpopulations

**FIGURE 4:**
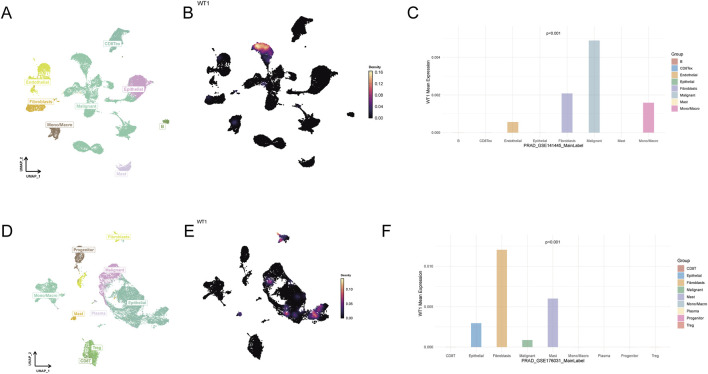
Single-cell mapping of WT1 across prostate cancer cellular compartments. **(A)** UMAP embedding of GSE141445 with major cell populations annotated using the original labels/marker genes. **(B)** WT1 signal on the GSE141445 UMAP is recovered and visualized using Nebulosa kernel density estimation to mitigate dropout effects. **(C)** Cell type-resolved mean WT1 expression in GSE141445 is summarized; group differences are evaluated with the Kruskal–Wallis rank-sum test (p value shown). **(D)** UMAP embedding of GSE176031 with annotated cell populations. **(E)** Nebulosa-based visualization of WT1 expression on the GSE176031 embedding. **(F)** Mean WT1 expression across annotated cell types in GSE176031 with significance assessed by the Kruskal–Wallis rank-sum test (p value shown).

To define the cellular origin of WT1 in prostate cancer, two independent scRNA-seq datasets (GSE141445 and GSE176031) were analyzed. UMAP embeddings delineated major malignant, epithelial, stromal, and immune compartments (Figures 4A,D). Given the sparsity typical of scRNA-seq, Nebulosa-based kernel density estimation was applied to visualize WT1 expression more robustly, revealing concentrated WT1 signal in malignant and stromal clusters (Figures 4B,E). Quantitative comparisons confirmed substantial heterogeneity of WT1 across cell types in both cohorts (Kruskal–Wallis p < 0.001). In GSE141445, WT1 expression was highest in malignant cells and fibroblasts, with additional signal in monocytes/macrophages, whereas lymphoid populations showed minimal expression (Figure 4C). In GSE176031, WT1 was most enriched in fibroblasts and mast cells, followed by epithelial cells, while malignant and T-cell subsets displayed comparatively lower levels (Figure 4F).

### 3.5 Spatial transcriptomics identifies region-restricted WT1 hotspots coupled to local cell-type composition

**FIGURE 5:**
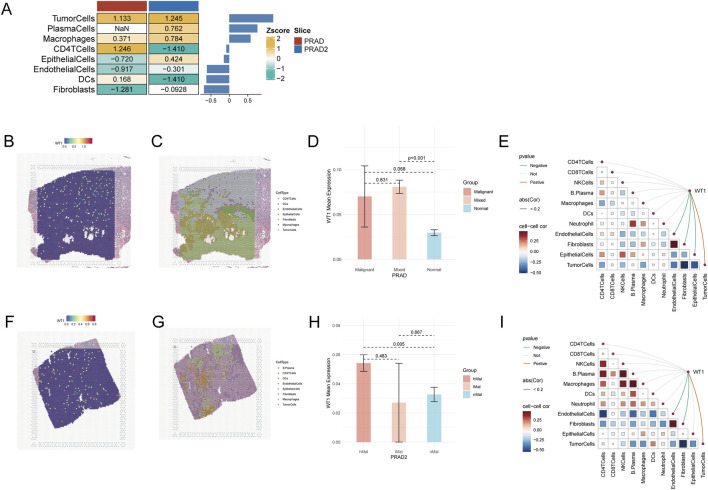
Spatially resolved WT1 distribution and microenvironmental associations in Visium PRAD sections. **(A)** Z-score–standardized WT1 expression is summarized across dominant cell-type microregions (spots assigned by the highest inferred cell-type proportion) in two prostate adenocarcinoma sections (PRAD and PRAD2). **(B,F)** SpatialFeaturePlot illustrates WT1 expression in PRAD **(B)** and PRAD2 **(F)**. **(C,G)** SpatialPlot depicts dominant microregion annotations for PRAD **(C)** and PRAD2 **(G)**. **(D)** WT1 levels are compared among malignant, mixed, and normal regions in PRAD as defined by malignant-cell proportion; Wilcoxon rank-sum test p values are shown. **(E)** Spearman correlations among inferred cell-type contents and between WT1 expression and cell-type contents are visualized using linkET for PRAD, with colors indicating correlation strength/direction and links denoting significant WT1–cell-type associations. **(H)** Region-wise WT1 comparisons in PRAD2 are shown for high-malignancy (hMal), low-malignancy (lMal), and non-malignancy (nMal) groups (Wilcoxon rank-sum tests; p values indicated). **(I)** Spearman correlation analysis for PRAD2 is displayed as in **(E)**.

WT1 distribution within tissue architecture was evaluated using two prostate adenocarcinoma Visium sections (PRAD and PRAD2) from the Sparkle database. Spots were annotated by dominant cell-type composition, and WT1 expression was summarized across microregions using Z-score standardization to enable cross-section comparison (Figure 5A). In both sections, WT1 reached its highest levels in tumor-cell-dominant microregions; enrichment among immune/stromal microregions differed between sections, including elevated WT1 in CD4 T-cell microregions in PRAD and in macrophage/plasma microregions in PRAD2 (Figure 5A). SpatialFeaturePlot further indicated focal WT1 hotspots co-localizing with malignant-dominant areas (Figures 5B–G). Region-stratified comparisons in PRAD showed higher WT1 levels in malignant or mixed regions relative to normal regions (Mixed vs. Normal p < 0.001; Malignant vs. Normal p = 0.068; Malignant vs. Mixed p = 0.831) (Figure 5D). In PRAD2, WT1 expression was significantly increased in high-malignancy regions compared with non-malignant regions (hMal vs. nMal p = 0.005), whereas other comparisons were not significant (Figure 5H). At spot level, Spearman correlation analysis demonstrated that WT1 was positively correlated with tumor-cell content and negatively correlated with endothelial and fibroblast content in both sections (Figures 5E,I), supporting preferential WT1 localization to malignant regions with reduced vascular/stromal signatures.

### 3.6 Structure-guided docking provides a testable hypothesis for spironolactone engagement with WT1

**FIGURE 6:**
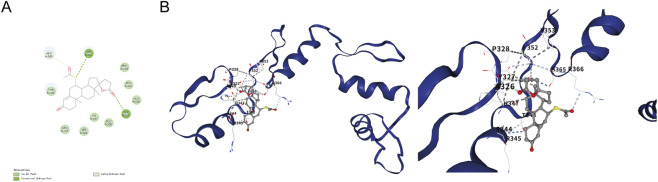
Structural docking suggests a plausible WT1 interaction mode for Spironolactone. **(A)** A 2D interaction map summarizes predicted hydrogen-bonding and hydrophobic contacts between Spironolactone and WT1 residues, including Tyr327 and Ala326. **(B)** A 3D rendering of the docked complex depicts Spironolactone positioned within a hydrophobic pocket of the WT1 structural domain.

To explore whether spironolactone could plausibly engage WT1, molecular docking was performed as a hypothesis-generating analysis. Predicted poses suggested that spironolactone may be accommodated within a WT1 pocket and could form potential hydrogen-bond and hydrophobic contacts with residues including Tyr327, Ala326, Ser344, Arg345, Glu350, and His347 (Figures 6A,B). As docking does not constitute experimental evidence of binding or functional modulation, these observations should be interpreted cautiously and require biochemical and pharmacological validation.

### 3.7 *In vitro* baseline profiling confirms WT1 suppression in PCa cell lines and validates robust overexpression

**FIGURE 7:**
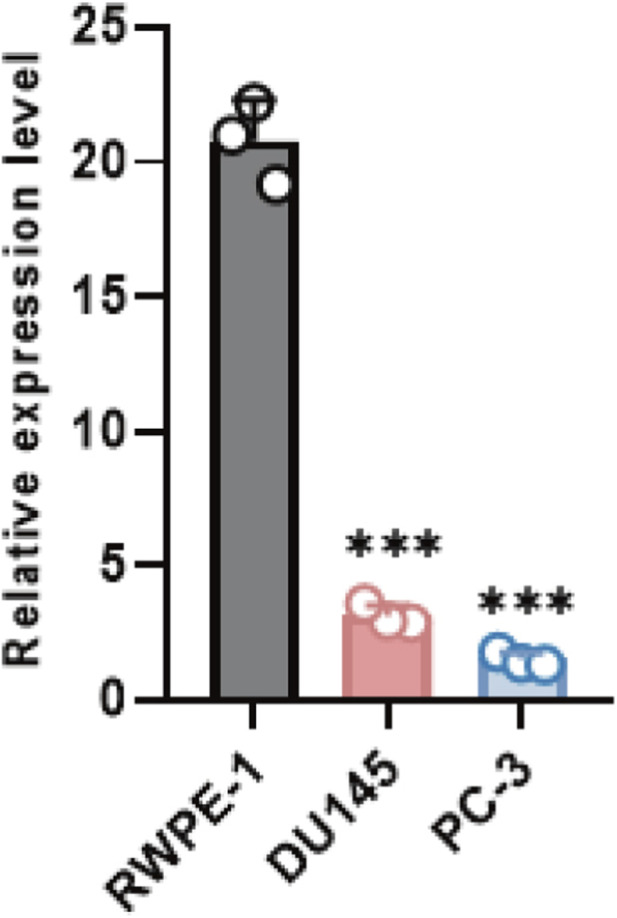
Baseline WT1 expression in normal and malignant prostate cell lines. Relative WT1 mRNA abundance was measured by quantitative real-time PCR in RWPE-1, DU145, and PC-3 cells, indicating marked downregulation in DU145 and PC-3 versus RWPE-1. Values are reported as mean ± SD (n = 3 independent biological replicates). Statistical significance was assessed using an unpaired two-tailed t-test. ***P < 0.001 vs. RWPE-1.

**FIGURE 8:**
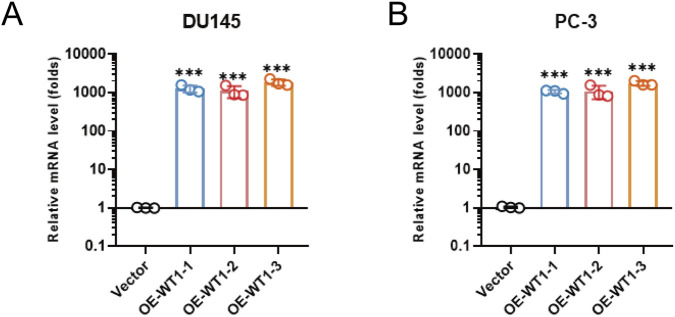
qRT-PCR validation of WT1 overexpression in prostate cancer models. **(A)** DU145 cells. **(B)** PC-3 cells. WT1 overexpression was introduced using three independent constructs (OE-WT1-1, OE-WT1-2, OE-WT1-3), and WT1 mRNA levels were quantified by quantitative real-time PCR relative to vector control. Data are presented as mean ± SD (n = 3 independent biological replicates). Statistical significance was determined using an unpaired two-tailed t-test. ***P < 0.001 vs. Vector.

WT1 mRNA abundance was quantified in the normal prostate epithelial cell line RWPE-1 and in prostate cancer cell lines DU145 and PC-3. qRT-PCR indicated that WT1 expression was substantially higher in RWPE-1, whereas DU145 and PC-3 exhibited marked downregulation, at levels approximately one-tenth or less of normal cells (Figure 7), consistent with the database-based findings. Three WT1-overexpressing derivatives (OE-WT1-1, OE-WT1-2, OE-WT1-3) were then established in both DU145 and PC-3 cells, using vector-transfected cells as controls. qRT-PCR verification showed that WT1 mRNA levels in overexpression groups were hundreds to nearly a thousand-fold higher than controls (Figures 8A,B), confirming robust construction of the WT1 overexpression system for downstream functional analyses.

### 3.8 WT1 re-expression suppresses short-term growth and long-term clonogenic outgrowth

**FIGURE 9:**
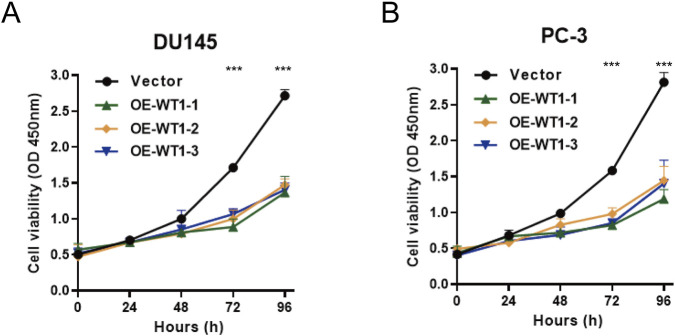
Growth suppression following WT1 overexpression measured by CCK-8. **(A)** DU145 cells. **(B)** PC-3 cells. Cell viability was tracked by CCK-8 at 0, 24, 48, 72, and 96 h in WT1-overexpressing groups (OE-WT1-1, OE-WT1-2, OE-WT1-3) and vector controls, with reduced OD450 values indicating inhibited proliferation upon WT1 upregulation. Results are shown as mean ± SD (n = 3 independent biological replicates). Statistical significance was determined using an unpaired two-tailed t-test. *p < 0.05, **p < 0.01, ***p < 0.001 vs. Vector.

**FIGURE 10:**
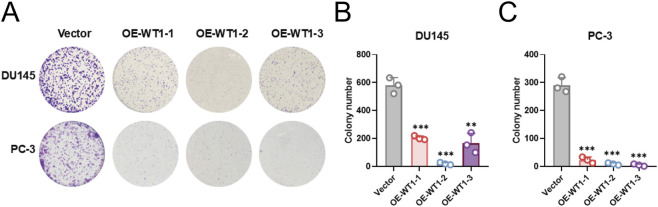
WT1 overexpression compromises clonogenic outgrowth in prostate cancer cells. **(A)** Representative colony formation images for DU145 and PC-3 cells transfected with OE-WT1-1, OE-WT1-2, OE-WT1-3 or vector control. **(B)** Colony counts for DU145. **(C)** Colony counts for PC-3. WT1 overexpression reduced colony numbers in both cell lines, consistent with impaired long-term proliferative capacity. Data are presented as mean ± SD (n = 3 independent biological replicates). Statistical analysis used an unpaired two-tailed t-test. **P < 0.01, ***P < 0.001 vs. Vector.

Cell proliferation was assessed by CCK-8. In both DU145 and PC-3 control groups, cell viability increased continuously over time; in contrast, all three WT1-overexpressing lines showed pronounced growth suppression, particularly at 72 and 96 h, reflected by reduced OD450 values compared with controls (Figures 9A,B). These data indicate that WT1 upregulation inhibits *in vitro* proliferation of prostate cancer cells. Colony formation assays corroborated this inhibitory effect. WT1-overexpressing DU145 and PC-3 cells formed fewer colonies than controls, with OE-WT1-2 exhibiting the strongest suppression, virtually abolishing colony formation (Figure 10A). Quantification confirmed significant reductions in colony numbers for all three overexpression strains relative to controls (Figures 10B,C), indicating impaired long-term proliferative and clonogenic potential following WT1 upregulation.

### 3.9 WT1 restoration diminishes migratory capacity across prostate cancer models

**FIGURE 11:**
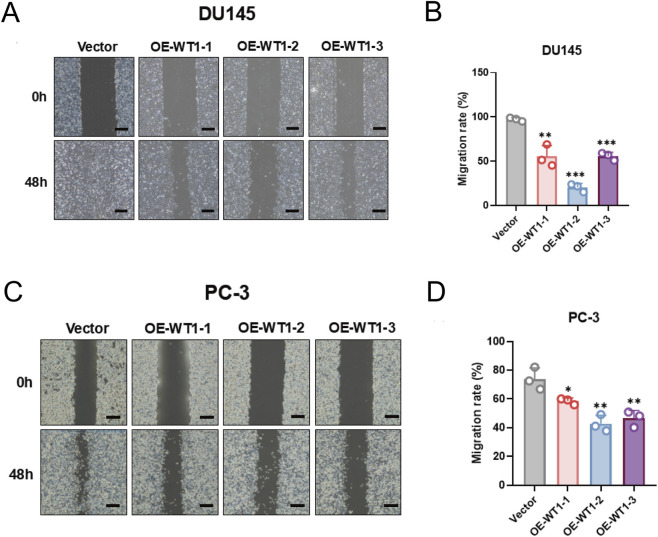
Reduced migratory behavior in WT1-overexpressing prostate cancer cells. **(A)** Representative wound-healing images of DU145 cells at 0 h and 48 h following transfection with OE-WT1-1, OE-WT1-2, OE-WT1-3 or vector control (Scale bar: 100 μm). **(B)** Migration rates quantified by wound-closure percentage at 48 h. WT1 overexpression decreased DU145 wound closure. Values are mean ± SD (n = 3 independent biological replicates); significance was tested with an unpaired two-tailed t-test. **P < 0.01, ***P < 0.001 vs. Vector. **(C)** Representative wound-healing images of PC-3 cells at 0 h and 48 h under the same conditions (Scale bar: 100 μm). **(D)** Migration rates at 48 h, showing impaired wound closure in WT1-overexpressing PC-3 cells. Data are mean ± SD (n = 3 independent biological replicates). Statistical significance was calculated using an unpaired two-tailed t-test. *P < 0.05, **P < 0.01 vs. Vector.

Migration was evaluated using scratch wound healing assays. In DU145 cells, control wounds were nearly fully closed at 48 h, whereas closure was clearly delayed in all three WT1-overexpressing groups; the OE-WT1-2 group retained a broad unhealed gap (Figure 11A). Quantitative analysis likewise demonstrated a marked reduction in migratory capacity with WT1 overexpression (Figure 11B). To assess generality, scratch assays were repeated in PC-3 cells. Similar to DU145, near-complete closure occurred in controls at 48 h, while wound healing remained notably impaired in OE-WT1-1, OE-WT1-2, and OE-WT1-3 groups (Figure 11C). Quantitative results showed significantly lower migration rates in all three WT1-overexpressing PC-3 lines, with OE-WT1-2 and OE-WT1-3 displaying the strongest inhibition (Figure 11D). Together, these results support a broad anti-migratory effect of WT1 across different prostate cancer cell lines.

### 3.10 WT1 upregulation activates apoptotic programs in DU145 and PC-3 cells

**FIGURE 12:**
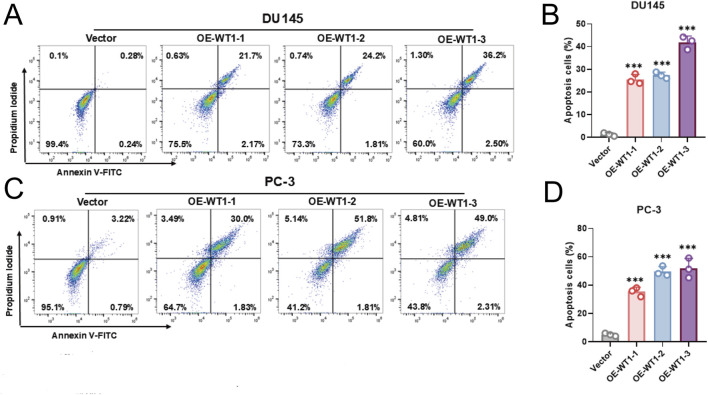
WT1 upregulation increases apoptotic fractions in prostate cancer cells. **(A)** Annexin V–FITC/PI flow cytometry profiles of DU145 cells transfected with OE-WT1-1, OE-WT1-2, OE-WT1-3 or vector control. **(B)** Quantification of DU145 apoptosis (early + late, %). **(C)** Annexin V–FITC/PI flow cytometry profiles of PC-3 cells under matched conditions. **(D)** Quantification of PC-3 apoptosis. WT1 overexpression elevated apoptotic populations in both DU145 and PC-3. Data are presented as mean ± SD (n = 3 independent biological replicates). Comparisons used an unpaired two-tailed t-test. ***P < 0.001 vs. V.ector.

Apoptosis was quantified by Annexin V-FITC/PI staining followed by flow cytometry in DU145 and PC-3 cells. Control populations displayed negligible apoptosis with minimal early and late apoptotic fractions. In contrast, all three WT1-overexpressing DU145 lines showed markedly increased apoptosis, with total apoptotic rates rising to approximately 30%–40%, most prominently in the OE-WT1-3 group (Figures 12A,B). A similar pattern was observed in PC-3 cells, where apoptotic rates increased to 40%–60% across the three overexpression strains, each significantly higher than the control group (Figures 12C,D). These findings indicate that WT1 upregulation promotes apoptosis and thereby contributes to suppression of prostate cancer cell growth.

## 4 Discussion

Therapeutic resistance remains the principal barrier to durable disease control in advanced prostate cancer, where progression to castration-resistant disease is frequently accompanied by an immune context that is difficult to therapeutically exploit. Within this clinical landscape, identifying regulators that simultaneously reflect tumor state and microenvironmental engagement is a persistent unmet need. WT1 is particularly challenging in this regard: although classically linked to tumor suppression in nephroblastoma, its role in adult solid tumors—including prostate cancer—has been reported as both oncogenic and protective depending on context. By integrating drug-target nomination with bulk, single-cell, and spatial transcriptomic evidence alongside functional perturbation, we define a WT1-associated biological state in PRAD and outline how this state could intersect with a spironolactone-centered repurposing hypothesis.

Across patient-derived datasets, elevated WT1 expression was consistently associated with features typically aligned with tumor restraint. WT1-high tumors exhibited signatures of immune microenvironment activation—most notably increased infiltration signals for anti-tumor lymphocyte populations—alongside broad shifts in metabolic and signaling programs. In parallel, restoration of WT1 expression in prostate cancer cell lines attenuated malignant behaviors and promoted apoptosis, supporting a context-dependent tumor-suppressive role. Taken together, these results suggest that WT1 is not merely a passive marker but may participate in regulatory programs that couple tumor-intrinsic state with immune surveillance.

WT1 was initially characterized as a tumor suppressor in nephroblastoma (Hirahata et al., 2025; Nian et al., 2025), yet subsequent work has emphasized its complex and sometimes opposing roles in adult cancers. Previous studies have indicated that WT1 can be aberrantly upregulated in multiple malignancies and may support tumor growth, survival, and adverse clinical outcomes (Hirahata et al., 2025; Nian et al., 2025). In acute leukemia and lung cancer, for example, heightened WT1 expression has been linked to proliferative signaling, resistance to apoptosis, and metastatic traits, coinciding with poorer prognosis (Hirahata et al., 2025). Within prostate cancer, earlier reports have also suggested an oncogenic contribution, including observations of higher WT1 levels in aggressive tumors relative to benign tissues (Hirahata et al., 2025; Nian et al., 2025). Mechanistically, prior literature has associated WT1 with reduced E-cadherin expression and induction of epithelial–mesenchymal transition (EMT), promoting migration and invasion (Brett et al., 2013). Additional studies have implicated WT1 in angiogenic remodeling through upregulation of factors such as VEGF, potentially facilitating neovascularization and progression (Nian et al., 2025). Collectively, these findings have supported a tumor-promoting interpretation of WT1 in prostate cancer biology (Brett et al., 2013; Nian et al., 2025).

In contrast, our analyses highlight an alternative facet of WT1 biology in clinical prostate cancer: higher WT1 expression tracked with immune activation, favorable disease associations, and anti-malignant functional effects *in vitro*. This context-dependence may stem from the complex isoform biology of WT1 (e.g., +KTS/-KTS variants), which dictates its affinity for DNA versus RNA and thus its regulatory output. Furthermore, the interplay between WT1 and Androgen Receptor (AR) signaling is critical; while we observed no significant difference in WT1 expression based on AR mutation status, previous studies suggest that WT1 can act as an AR co-factor. It is plausible that Spironolactone, as an AR antagonist, may indirectly influence this axis, shifting the balance from oncogenic to tumor-suppressive WT1 programs.

The immune and metabolic differences observed between WT1 strata provide further biological support for this interpretation. GSVA indicated that WT1-high tumors were enriched for immune-related programs, including antigen presentation and T cell–associated responses, consistent with a more inflamed or immunologically engaged microenvironment. In parallel, WT1-high tumors displayed coordinated shifts in metabolic gene sets, suggesting metabolic remodeling accompanying immune activation. This observation aligns with recent studies indicating that metabolic reprogramming functions as a critical driver of immune escape in the tumor microenvironment (Cong et al., 2025). Although our data are not sufficient to establish metabolic causality, the observed pattern is compatible with a state that favors oxidative programs and reduced glycolytic dependence, potentially limiting proliferative capacity; this mechanistic link will require direct functional validation. These observations are also consistent with prior reports implicating WT1 in differentiation-associated programs and broader cellular homeostasis (Hirahata et al., 2025).

Independent immune deconvolution using the TIP framework further reinforced the association between WT1 and an active cancer–immunity cycle. TIP integrates stage-specific indicators across tumor antigen release, immune recruitment, and effector function (Ogasawara, 2024). In WT1-high tumors, multiple parameters—including effector T cell infiltration and cytotoxic effector readouts—were elevated relative to WT1-low tumors, consistent with enhanced immune recognition and elimination capacity. Prior studies have indicated that WT1 can function as a tumor antigen capable of eliciting clinically meaningful immune responses; notably, improved disease control has been reported in patients mounting strong responses to WT1-directed vaccine strategies (Cheever et al., 2009; Ogasawara, 2024). In this light, WT1-high tumors may represent a more immunogenic subset that is intrinsically more visible to immune surveillance, providing a plausible explanation for their more favorable clinical associations. Importantly, this model does not require WT1 to act solely through tumor-intrinsic transcriptional control; rather, it suggests that WT1-linked states may integrate intrinsic differentiation/proliferation constraints with extrinsic immune pressure.

A central objective of the present work was to explore how spironolactone might intersect with WT1-centered biology. Network-based target prioritization and molecular docking were used here as hypothesis-generating strategies rather than definitive evidence of physical binding. The docking results should be interpreted as a predictive model suggesting structural possibility, which requires future biophysical validation, especially given that WT1 is a nuclear transcription factor and not a canonical small-molecule target. Nonetheless, the docking results support structural plausibility for an interaction interface, and the network context suggests additional routes by which spironolactone could influence WT1-related programs indirectly. One feasible model is that spironolactone’s anti-androgenic activity perturbs AR-driven transcriptional states that co-vary with WT1 expression or downstream pathway activity. Another is that spironolactone modulates upstream regulators or inflammatory mediators identified within the prioritized network (including TP53-associated or immune-linked nodes), thereby reshaping WT1-adjacent regulatory programs without requiring direct engagement of WT1. These possibilities remain speculative but experimentally tractable.

Taken together, our results reframe WT1 as a multi-omics-defined, immune-associated marker in prostate cancer that aligns with tumor-restraining phenotypes and exerts measurable anti-malignant effects when restored *in vitro*. More broadly, the study illustrates a pragmatic repurposing pipeline in which network pharmacology prioritizes candidates, bulk and single-cell modalities clarify clinical and cellular context, and spatial mapping links molecular signals to tissue architecture. Within this framework, the proposed spironolactone–WT1 axis provides a concrete direction for mechanistic and pharmacological validation and may support biomarker-guided stratification strategies in genitourinary oncology.

Several limitations of this study must be acknowledged. **First**, regarding the validation of the Spironolactone-WT1 axis, our study focused on identifying WT1 as a downstream target via network pharmacology and validating WT1’s functional role via genetic overexpression. We did not perform direct Spironolactone treatment assays or biophysical binding experiments (e.g., SPR or CETSA) in this scope. While molecular docking suggests a potential interaction, the direct modulation of WT1 protein levels by Spironolactone remains to be experimentally characterized in future work. **Second**, our functional validations relied primarily on mRNA-level quantification. Although qRT-PCR confirmed robust overexpression, future studies should include Western blotting and immunofluorescence to rigorously assess WT1 protein abundance and nuclear localization. **Third**, regarding the safety profile, we did not evaluate the effects of WT1 overexpression in normal prostate epithelial cells (e.g., RWPE-1) in this study, which is an important consideration for potential toxicity. **Finally**, our spatial transcriptomic analysis was limited to two Visium sections. Given the high heterogeneity of prostate cancer, these spatial findings should be interpreted as exploratory. Future investigations with larger cohorts are needed to statistically generalize these microenvironmental patterns.

## 5 Conclusion

In summary, this study establishes a multi-dimensional computational and experimental framework that supports the repositioning of Spironolactone (SPI) for Prostate Adenocarcinoma (PRAD) management. By integrating network pharmacology with multi-omics profiling, we identified Wilms Tumor 1 (WT1) as a pivotal, immune-correlated biomarker that is significantly downregulated in malignant tissues and associated with favorable disease-free survival. Our findings suggest that SPI may exert anti-tumor effects potentially involving the WT1 signaling axis. While our data support WT1 as a functional tumor suppressor, the direct pharmacological engagement of WT1 by SPI remains a hypothesis warranting further mechanistic investigation. Furthermore, *in vitro* restoration of WT1 effectively attenuated malignant phenotypes—suppressing proliferation and migration while inducing apoptosis—thereby validating WT1 as a functional tumor suppressor in this context. Collectively, these data provide a compelling rationale for further pharmacological validation and suggest that the SPI-WT1 axis could serve as a novel therapeutic target for precision oncology in genitourinary cancers.

## Data Availability

The datasets presented in this study can be found in online repositories. The names of the repository/repositories and accession number(s) can be found in the article/supplementary material.
